# Fast Feature-Preserving Approach to Carpal Bone Surface Denoising

**DOI:** 10.3390/s18072379

**Published:** 2018-07-21

**Authors:** Ibrahim Salim, A. Ben Hamza

**Affiliations:** Concordia Institute for Information Systems Engineering, Concordia University, Montreal, QC H3G 1M8, Canada; ibrahim_salim1976@yahoo.com

**Keywords:** mesh denoising, kernel similarity, matrix balancing, carpal bones

## Abstract

We present a geometric framework for surface denoising using graph signal processing, which is an emerging field that aims to develop new tools for processing and analyzing graph-structured data. The proposed approach is formulated as a constrained optimization problem whose objective function consists of a fidelity term specified by a noise model and a regularization term associated with prior data. Both terms are weighted by a normalized mesh Laplacian, which is defined in terms of a data-adaptive kernel similarity matrix in conjunction with matrix balancing. Minimizing the objective function reduces it to iteratively solve a sparse system of linear equations via the conjugate gradient method. Extensive experiments on noisy carpal bone surfaces demonstrate the effectiveness of our approach in comparison with existing methods. We perform both qualitative and quantitative comparisons using various evaluation metrics.

## 1. Introduction

Recent advances in 3D scanning technology have led to the increasing use of 3D models in many fields, including the entertainment industry, archaeology, computer vision, and medical imaging. These models are usually captured in the form of point clouds or polygonal meshes [1], but they are often corrupted by noise during the data acquisition stage. The main problem with 3D shape denoising is how we can distinguish between noise and features, especially sharp surface features. To ensure the development of high-quality 3D shapes for use in downstream applications, it is important to develop effective surface denoising techniques to remove inevitable noise in the measurements [2,3,4,5,6,7,8].

In recent years, a plethora of techniques have been proposed to tackle the 3D surface denoising problem. Generally, surface denoising methods can be classified into two major categories: isotropic and anisotropic. The former techniques filter the noisy data independently of direction, while the latter methods modify the diffusion equation to make it nonlinear or anisotropic in order to preserve the sharp features of a 3D mesh surface. The simplest surface denoising method is the Laplacian flow which repeatedly and simultaneously adjusts the location of each mesh vertex to the geometric center of its neighboring vertices [2].

Most surface denoising methods are adopted from the image processing literature [9,10,11,12], including the use of mean, median, and bilateral filters. In particular, bilateral filtering has been used extensively in image processing applications, due, in large part, to its good performance in smoothing noisy images while preserving edges. The bilateral filter takes into account the variation in image intensity by replacing the intensity value at a pixel by a weighted average of the intensity values from neighboring pixels. Although these filters have been successfully applied to image denoising, it is, however, not straightforward to apply them directly to graph-structured data. Fleishman et al. [5] proposed a bilateral mesh denoising approach that filters each mesh vertex in the normal direction using local neighborhoods. Zheng et al. [8] applied the bilateral normal filter in a local iterative and a global non-iterative scheme for anisotropic denoising. Sun et al. [13] introduced a two-step mesh denoising framework. In the first step, the noisy face normals are filtered iteratively by weighted averaging of neighboring face normals. In the second step, the mesh vertex positions are iteratively updated based on the denoised face normals. Huang and Uscher proposed a multiscale anisotropic Laplacian (MSAL) model [14], which employs the anisotropic Laplacian operator combined with a roughness scale and yields significantly better results than the anisotropic Laplacian model and the bilateral filter. Ouafdi et al. [15] introduced a probabilistic mesh denoising method by performing anisotropic averaging of neighboring vertices weighted by a Riemannian metric. Zhang et al. [16] presented a guided mesh normal filtering framework by constructing the guidance for joint bilateral filtering of geometry signals using a two-step process. Joint bilateral filtering is applied to the face normals, followed by updating the mesh vertices to agree with the denoised face normals. More recently, Yadav et al. [17] proposed a two-stage mesh denoising approach using robust statistics. In the first stage, the face normals are filtered via bilateral normal filtering using Tukey’s bi-weight as a similarity function. In the second stage, the mesh vertex positions are updated using edge-to-face normal orthogonality constraints along with differential coordinates.

On the other hand, image/surface denoising via graph signal processing techniques has received considerable attention in recent years [12,18,19]. A graph-based approach to image denoising and deblurring was introduced in [12] using a data-adaptive objective function derived from a normalized graph Laplacian. Chung et al. [19] used the graph Laplacian to construct the discrete version of heat kernel smoothing on graph-structured data obtained by binary segmentation of the computed tomography of human lung data. Also, Chung et al. [20] introduced a heat kernel regression approach to surface smoothing using the Laplace–Beltrami eigenfunctions which are obtained by solving a generalized eigenvalue problem. Such an approach can, however, be prohibitively expensive, especially when the problem size is large (i.e., large matrices). Another issue with spectral approaches is how to select the appropriate number of eigenvalues and associated eigenfunctions to be retained.

Motivated by the good performance of the similarity-based image denoising framework proposed in reference [12], we introduce a simple, yet effective, feature-preserving approach to 3D mesh denoising. The proposed method employs a normalized mesh Laplacian, which is defined in terms of a data-adaptive kernel similarity matrix in conjunction with matrix balancing. We formulate our surface denoising framework as a constrained minimization problem, which can be solved efficiently using the conjugate gradient (CG) method. Our approach can remove noise effectively while preserving the nonlinear features of surfaces, such as curved surface regions, sharp edges, and fine details. While our proposed framework is general enough to be applied to any problem involving surface denoising, the primary focus of this work is on noise removal from carpal bone surfaces. Further, recovering high quality surfaces from noisy carpal bone surfaces is a fundamental problem in computational anatomy and biomechanics and is of paramount importance to the diagnosis of wrist pathologies, such as arthritis. Our main contributions may be summarized as follows:

- We introduce a mesh denoising approach using a data-adaptive kernel similarity matrix in conjunction with matrix balancing.
- We formulate the proposed framework as a constrained minimization problem and solve it iteratively using the conjugate gradient method.
- Our experimental results show superior performance of the proposed framework over existing mesh denoising methods.

The rest of this paper is organized as follows. In Section 2, we briefly recall some basic concepts of geometry processing, followed by a general formulation of the surface denoising problem in the graph signal processing setting. In Section 3, we present the main building blocks of our method, and discuss, in detail, the algorithmic steps. In Section 4, we present experimental results to demonstrate the competitive performance of our denoising approach on carpal bone surfaces. Finally, Section 5 concludes the paper and points out future work directions.

## 2. Problem Formulation

**Triangular mesh representation:** A 3D shape is usually modeled as a triangle mesh, M, whose vertices are sampled from a Riemannian manifold. A triangle mesh, M, may be defined as a graph, G=(V,E) or G=(V,T), where $V=\{v_{1},\ldots ,v_{n}\}$ is the set of vertices, $E=\{e_{ij}\}$ is the set of edges, and $T=\{t_{1},\ldots ,t_{m}\}$ is the set of triangles. Each edge, $e_{ij}=\left[v_{i},v_{j}\right]$, connects a pair of vertices, $\left\{v_{i},v_{j}\right\}$. Two distinct vertices, $v_{i},v_{j}\in V$, are adjacent (denoted by $v_{i}∼v_{j}$ or simply i∼j) if they are connected by an edge, i.e., $e_{ij}\in E$. The neighborhood of a vertex, $v_{i}$, is the set ${\overset{˚}{v}}_{i}=\left\{v_{j}\in V:v_{j}∼v_{i}\right\}$.

**Laplacian matrix of a weighted graph:** The graph, G, may be equipped with a nonnegative weight function, $\omega :V\times V\to R^{+}$, such that

(1) $$\omega_{ij}=\left\{\begin{array}{cc} \omega_{ii} & \mathrm{if}\;i=j\\ \omega_{ij} & \mathrm{if}\;i∼j\\ 0 & \mathrm{otherwise} \end{array}.\right.$$

The Laplacian matrix, $L=(\ell_{ij})$, of a weighted graph is defined as L=D−A, whose elements are given by

(2) $$\ell_{ij}=\left\{\begin{array}{cc} d_{i}-\omega_{ii} & \mathrm{if}\;i=j\\ -\omega_{ij} & \mathrm{if}\;i∼j\\ 0 & \mathrm{otherwise} \end{array},\right.$$

where $A=(w_{ij})$ is the weighted adjacency matrix, and $D=\mathrm{diag}(d_{1},\ldots ,d_{n})$ is the degree matrix with $d_{i}=\sum_{j∼i}\omega_{ij}$ being the degree of vertex *i*. The normalized weighted Laplacian matrix, L, is defined as

(3) $$L=D^{-1/2}LD^{-1/2}=I-D^{-1/2}AD^{-1/2}.$$

**Figure 1:**
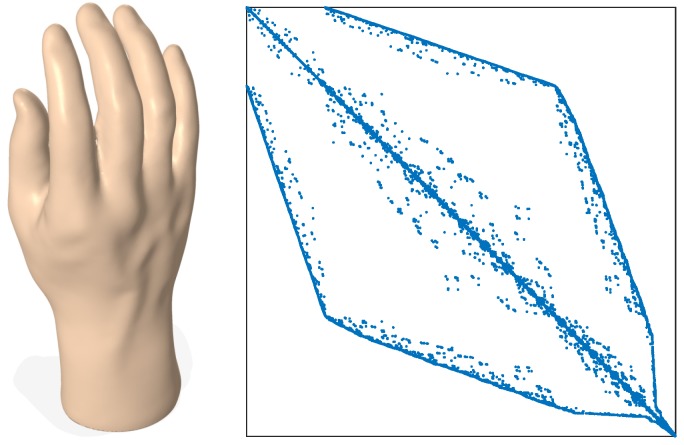
Hand model (**left**) and sparsity pattern plot of its weighted Laplacian matrix (**right**).

Figure 1 displays a 3D hand model and its weighted Laplacian matrix, with weights $\omega_{ij}=∥v_{i}-v_{j}∥$, where ∥·∥ denotes the Euclidean norm. The sparsity pattern (or support) of $L=(\ell_{ij})$ is the set of indices, ij, with $\ell_{ij}\neq 0$.

The Laplacian matrix may be viewed as an operator defined on the space of graph signals, u:V→R, as follows:

(4) $$Lu\left(i\right)=\sum_{i∼j}\omega_{ij}\left(u\left(i\right)-u\left(j\right)\right),\;\mathrm{for}\;\mathrm{all}\;i\in V.$$

In other words, Lu(i) is the sum of the weighted differences between the value of the graph signal, *u*, at vertex *i* and the values at the neighboring vertices.

Since |V|=n, we may represent any graph signal, u:V→R, as a column vector, $u=\left(u\left(i\right)\right)\in R^{n}$, with the *i*th element, u(i). Thus, the quadratic form of the signal, u, with respect to the Laplacian matrix can be expressed as

(5) $$u^{⊺}Lu=\sum_{i∼j}\omega_{ij}{\left(u\left(i\right)-u\left(j\right)\right)}^{2},$$

which shows that if the weights are symmetric, then the Laplacian matrix is symmetric positive semi-definite. So the action of the Laplacian on a signal may be viewed as measuring the smoothness of that signal across the edges in the mesh.

### Mesh Denoising Model

In all real applications, measurements are usually perturbed by noise. In the course of acquiring, transmitting or processing a 3D model, for example, the noise-induced degradation often yields a resulting graph signal observation model, and the most commonly used is the additive one,

(6) v=u+η,

where the observed graph signal, v, includes the original graph signal, u, and the random noise process, η, which is usually assumed to be Gaussian with zero mean and standard deviation σ.

Surface denoising refers to the process of recovering a 3D model contaminated by noise. The challenge of the problem of interest lies in recovering the graph signal, u, from the observed signal v, and furthering the estimation by making use of any prior knowledge/assumptions about the noise process η.

When considering the noise model (6), our goal may be succinctly stated as one of estimating the underlying graph signal, u, based on an observed signal, v, and/or any potential knowledge of the noise statistics to further regularize the solution. This yields the following fidelity-constrained optimization problem

(7) $$\begin{array}{ll} \min_{u} & R(u)\\ s.t. & {∥v-u∥}^{2}\leq \sigma^{2} \end{array}$$

where R is a given regularization functional, which often defines the particular emphasis on the features of the achievable solution. In other words, we want to find an optimal solution that yields the smallest value of the objective function among all solutions that satisfy the constraints. Using Lagrange’s theorem, the minimizer of (7) is given by

(8) $$\hat{u}=\arg\min_{u}\left\{{∥v-u∥}^{2}+\beta R\left(u\right)\right\},$$

where β is a non-negative regularization parameter, which is often estimated or chosen *a priori*. A critical issue, however, is the choice of the regularization functional, R, which is often driven by geometric arguments. A commonly used functional is the mesh Laplacian quadratic form defined as a (squared) weighted vector norm:

(9) $$R\left(u\right)={∥u∥}_{L}^{2}=u^{⊺}Lu.$$

## 3. Methods

**Figure 2:**
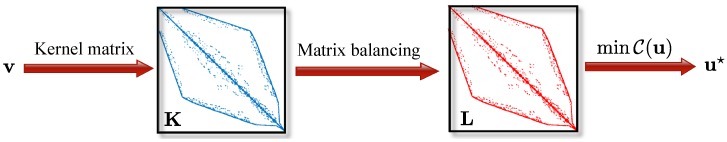
Flowchart of our proposed surface denoising method, where v is the noisy graph signal, and $u^{⋆}$ is the estimated signal.

In this section, we present the main components of the proposed surface denoising framework and describe, in detail, its algorithmic steps. The flowchart of our approach is illustrated in Figure 2.

**Kernel similarity:** Using the Gaussian kernel, we define the kernel weight matrix, $S=(s_{ij})$, as

(10) $$s_{ij}=\exp\left(-\frac{∥v_{i}-v_{j}{∥}^{2}}{2h^{2}}\right),$$

where $v_{i}$ is the *i*th vertex of the noisy mesh, $v_{j}$ are the neighboring vertices of $v_{i}$, and *h* is the bandwidth parameter of the Gaussian kernel. Each edge weight, $s_{ij}$, is a similarity measure whose value is large when *i* is closer to *j*. We define the kernel similarity weight matrix as follows:

(11) $$K=\frac{S+S^{⊺}}{2},$$

which is a symmetric, non-negative matrix. Further, all of its off-diagonal elements are positive.

**Sinkhorn matrix balancing:** Applying the Sinkhorn matrix balancing procedure [21] to the kernel similarity weight matrix, K, yields a symmetric non-negative doubly stochastic filtering matrix, W, given by

(12) $$W=C^{-1/2}KC^{-1/2},$$

where C is a diagonal scaling matrix [22]. It should be noted that since the filtering matrix, W, is doubly stochastic, its largest eigenvalue is equal to 1 with the associated eigenvector, $e=1/\sqrt{n}$, where 1 is a vector of all ones. In other words, the filtering matrix preserves the DC component of a graph signal (i.e., We=e).

**Normalized mesh Laplacian:** We define the normalized mesh Laplacian matrix as

(13) $$L=I-W=I-C^{-1/2}KC^{-1/2},$$

which is symmetric positive semi-definite. The Laplacian matrix, L, can be interpreted as a data-adaptive high-pass filter, enabling us to incorporate a variety of filters in the data term as well the regularization term.

**Figure 3:**
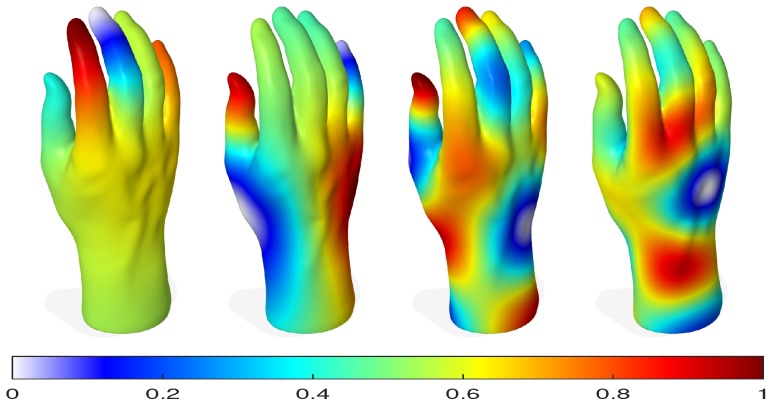
Visualization of four eigenvectors of the normalized mesh Laplacian matrix. From **left** to **right**: a 3D hand model Gouraud shaded and color-coded by the values of the second, eighth, fifteenth and twentieth eigenvectors.

From (13), it is easy to see that if λ is an eigenvalue of W, then 1−λ is an eigenvalue of L. In particular, 0 is an eigenvalue of L with the associated eigenvector, e. The eigenvalues of L may be viewed as graph frequencies. Moreover, the eigenvectors associated with the smallest eigenvalues have smooth oscillations and capture the large-scale properties of a shape well. As shown in Figure 3, the (non-trivial) eigenvectors of L encode important information about the global geometry of a shape. Notice that the eigenvectors associated with larger eigenvalues oscillate more rapidly. Blue regions indicate small eigenvector values and red regions indicate large values, while green and yellow regions are in between.

### 3.1. Surface Denoising Approach

We formulated our surface denoising framework as a constrained optimization problem by minimizing the following cost function

(14) $$\begin{array}{cc} C(u) & ={∥v-u∥}_{I+\alpha L}^{2}+\beta {∥u∥}_{L}^{2}\\ & ={\left(v-u\right)}^{⊺}\left(I+\alpha L\right)\left(v-u\right)+\beta u^{⊺}Lu, \end{array}$$

where v is the noisy graph signal and u is the estimated signal. The non-negative parameters, α and β, are often estimated or chosen *a priori*. Note that the first term is a weighted error between the input and its estimate, and minimizing such an error yields a solution as close as possible to the input. Minimizing the second term, on the other hand, yields a smooth solution. Further, I+αL is a symmetric, positive-definite matrix.

The cost function, C(u), can be minimized by finding its gradient and setting it to zero

(15) ∇C(u)=−2(I+αL)(v−u)+2βLu=0,

resulting in the following system of linear equations:

(16) $$\begin{array}{c} \left(I+(\alpha +\beta )L\right)u=\left(I+\alpha L\right)v. \end{array}$$

Since I+(α+β)L is a symmetric, positive-definite matrix, system (16) can be efficiently solved using iterative methods such as the CG method, which is a commonly used iterative algorithm for solving sparse systems of linear equations.

### 3.2. Algorithm

The objective of 3D mesh denoising is to remove noise while preserving features. Our proposed surface denoising approach consists of two major steps, as illustrated in Figure 2. In the first step, the normalized mesh Laplacian is computed using kernel similarity and matrix balancing. In the second step, a sparse system of linear equations is iteratively solved using the CG method. It should be noted that the proposed algorithm consists of both outer and inner iterations. The outer iterative process is used to compute the normalized mesh Laplacian, while the inner iterative process is employed to solve the constrained minimization problem. Algorithm 1 summarizes the main algorithmic steps of our approach.

**Algorithm 1** Feature-Preserving Mesh Denoising**Input** Noisy graph signal v 1:${\hat{u}}^{\left(0\right)}=v$2:k=0.3:**while** not converged **do**4:  Compute the kernel similarity weight matrix K from ${\hat{u}}^{\left(k\right)}$ using (10)–(11)5:  Apply Sinkhorn matrix balancing to K to get the diagonal matrix C6:  Compute the Laplacian matrix $L=I-C^{-1/2}{\mathrm{KC}}^{-1/2}$7:  Solve the linear system in (16) using conjugate gradient to estimate ${\hat{u}}^{\left(k+1\right)}$.8:  Set $u^{⋆}={\hat{u}}^{\left(k+1\right)}$9:  k=k+110:**end while**
**return**
$u^{⋆}$
**Output** Estimated signal $u^{⋆}$

## 4. Experiments

**Figure 4:**
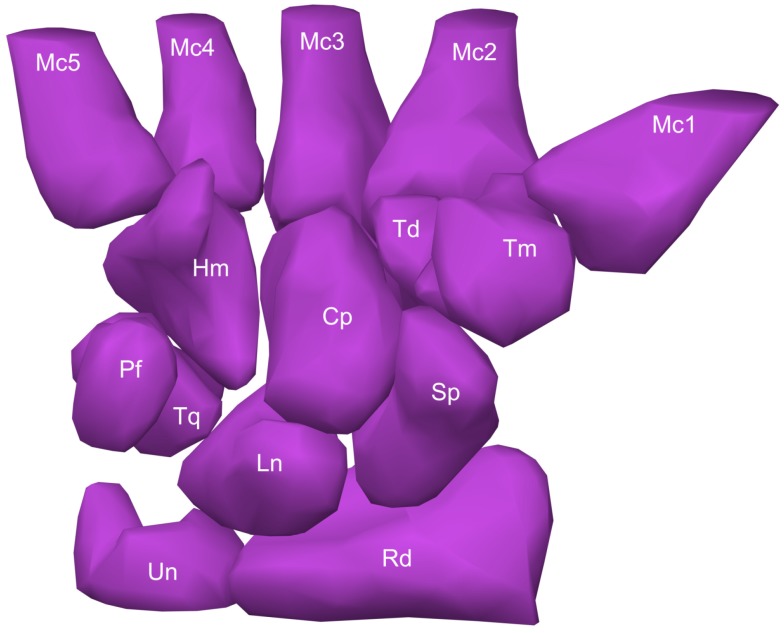
Carpal bone anatomy of a healthy male from a palmar view. The carpus consists of eight carpal bones which are arranged in proximal and distal rows. The proximal row contains the scaphoid (Sp), lunate (Ln), triquetrum (Tq), and pisiform (Pf), while the distal row contains the trapezium (Tm), trapezoid (Td), capitate (Cp), and hamate (Hm). The distal row adjoins the five metacarpals (Mc1-5) of the wrist. The radius (Rd) and ulna (Un) are also shown.

In this section, through extensive experiments, we evaluate the performance of our proposed mesh denoising approach on carpal bone surfaces [23]. As shown in Figure 4, the carpal bones of the right wrist in a healthy male are the capitate, hamate, lunate, pisiform, scaphoid, trapezium, trapezoid, and triquetrum. Since the trapeziometacarpal joint of the thumb is a common site for osteoarthritis, the first metacarpal bone is also considered in our analysis. The forearm’s radius and ulna bones, which support the many muscles that manipulate the bones of the hand and wrist, are also depicted in Figure 4.

**Implementation details:** All experiments were performed on a desktop computer with an Intel Core 2 Duo running at 3.40 GHz and 16 GB RAM, and the proposed mesh denoising algorithm was implemented in MATLAB. The parameters, α and β, were chosen as the inverse of the minimum and maximum of the mesh degree values, respectively (i.e., $\alpha =1/d_{min}$ and $\beta =1/d_{max}$). The kernel bandwidth parameter, *h*, was estimated using the median absolute deviation (MAD) as follows:

(17) $$h=1.4826\sum_{i=1}^{n}\underset{j∼i}{\mathrm{MAD}}\left(v_{i}-v_{j}\right),$$

where MAD(x)=median(∥x−median(∥x∥)∥) is a measure of spread that represents the expected absolute-error loss, and is robust to outliers.

**Baseline methods:** We compared the effectiveness of our proposed technique with several state-of-the-art approaches, including bilateral mesh denoising (BMD) [5], the multiscale anisotropic Laplacian (MSAL) method [14], guided mesh normal denoising (GMD) [16], and robust and high fidelity mesh denoising (RMD) [17].

### 4.1. Results

**Figure 5:**
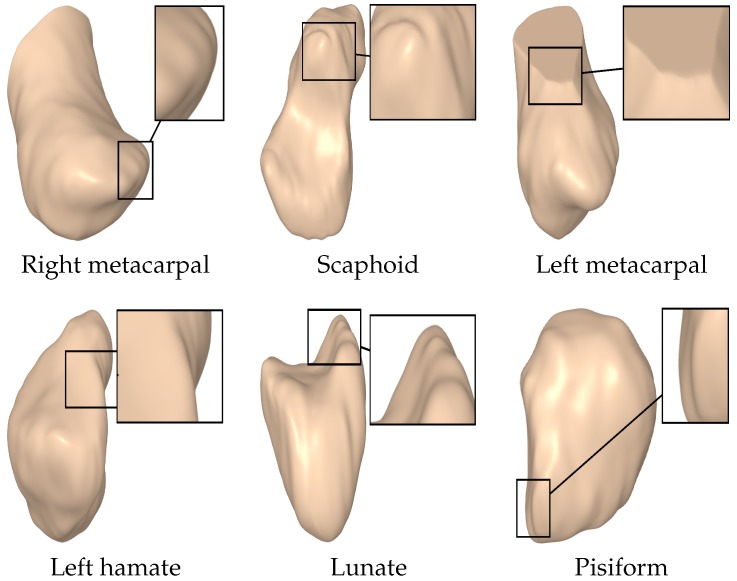
Carpal bone models.

We performed extensive experiments on various carpal bone surfaces, including the right metacarpal, scaphoid, left metacarpal, left hamate, lunate, and pisiform, as shown in Figure 5.

We generated the noisy carpal bone models by setting the standard deviations of the noise to 0.5$\bar{\ell }$ and 0.7$\bar{\ell }$ of the mean edge length $\bar{\ell }$, as given by

(18) $$\bar{\ell }=\frac{1}{\left|E\right|}\sum_{e_{ij}\in E}∥e_{ij}∥,$$

where $∥e_{ij}∥=∥v_{i}-v_{j}∥$ if i∼j, and $∥e_{ij}∥=0$ otherwise. More precisely, a vertex, $v_{i}$, of a noisy mesh is given by the additive random noise model:

(19) $$v_{i}=u_{i}+\sigma \left(\eta_{i}⊙n_{i}\right),$$

where $\eta_{i}$ are i.i.d. Gaussian random vectors (i.e., $\eta_{i}$ is a 3-dimensional vector containing pseudorandom values drawn from the standard normal distribution, N(0,1)), $n_{i}$ is the unit normal vector at the noise-free vertex, $u_{i}$, and ⊙ denotes the Hadamard product between two vectors (i.e., the elements of vector $\eta_{i}⊙n_{i}$ are obtained via element-by-element multiplication of vectors $\eta_{i}$ and $n_{i}$).

#### 4.1.1. Qualitative Comparison

**Figure 6:**
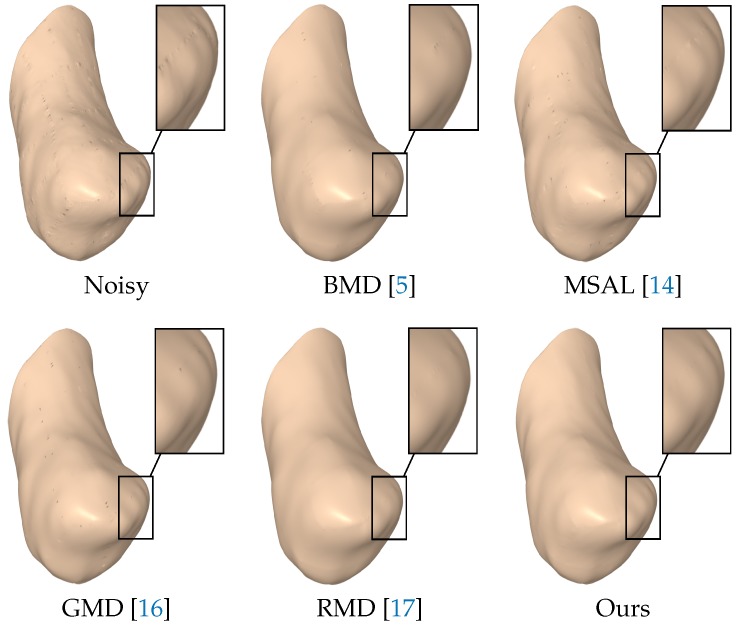
Surface denoising results of the noisy right metacarpal model corrupted by Gaussian noise with σ=0.5. The magnified views of denoised models show that our method outperformed the baselines in preserving the surface features.

**Figure 7:**
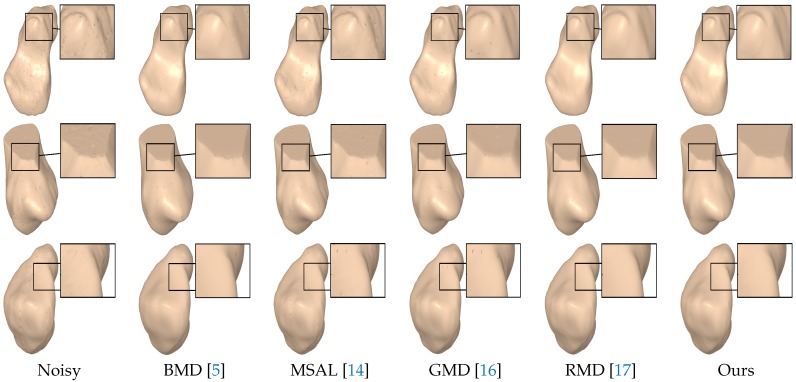
Surface denoising results for the noisy scaphoid, left metacarpal, and left hamate models. The noise standard deviation was set to σ=0.5.

The visual comparison was performed with the most prevalent methods of 3D mesh denoising, including BMD [5], MSAL [14], GMD [16] and RMD [17]. As shown in Figure 6, the noisy right metacarpal model was generated by adding a Gaussian noise with a standard deviation of σ=0.5 to the vertices of the ground truth mesh along the vertex normals. As can be seen, the output results of BMD, MSAL, GMD and RMD still contained a considerable amount of noise in some regions of the denoised model, while the proposed approach removed the noise well and, at the same time, preserved the surface detail. Figure 7 displays the denoising results on the noisy scaphoid, left metacarpal, and left hamate models with a noise standard deviation of σ=0.5, proportional to the mean edge length of the mesh. Notice again that the proposed approach preserved the edges well, while RMD tended to over-smooth the features. Further, the noise was mostly eliminated using our approach without affecting flat regions. Further, the sharp features were well preserved, as depicted in the enlarged views, which shows that the geometric structures and the fine details of the denoised carpal bone models were very well preserved.

**Figure 8:**
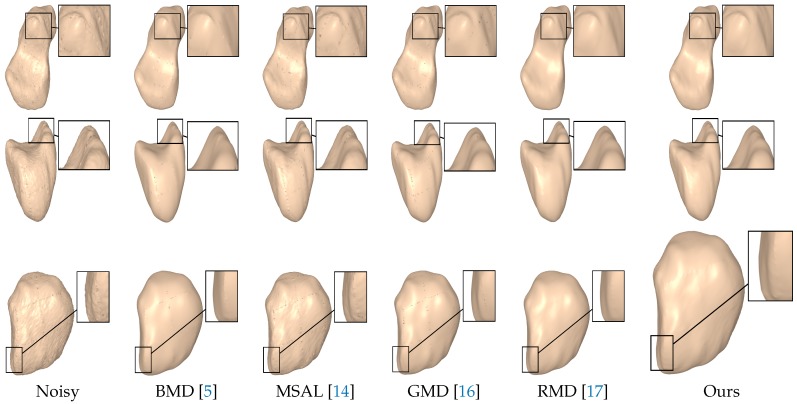
Surface denoising results for the noisy scaphoid, lunate, and pisiform models. The noise standard deviation was set to σ=0.7.

Figure 8 shows the denoising results of the noisy scaphoid, lunate, and pisiform models with a higher noise standard deviation, σ=0.7, proportional to the mean edge length of the mesh. As can be seen, RMD removed the noise relatively well but did not preserve the sharp features. The other baseline methods did not remove the noise well and also tended to over-smooth the sharp regions, while our approach effectively removed the noise without creating any edge flips. While RMD yielded comparable results to our approach, it did not, however, preserve edges with the same effectiveness.

In all the experiments, we observed that our approach was able to suppress noise while preserving important geometric features of the carpal bone surfaces in a fast and efficient manner. This better performance is, in fact, consistent with a large number of 3D models used for experimentation.

#### 4.1.2. Quantitative Comparison

To quantify the difference between the ground truth and estimated model, we used three different measures, namely, the mean orientation error metric, the face-normal error metric, and the face quality metric [17].

Let M=(V,T) and $\hat{M}=\left(\hat{V},\hat{T}\right)$ be the original and denoised models with vertex sets $V={\left\{v_{i}\right\}}_{i=1}^{n}$ and $\hat{V}={\left\{{\hat{v}}_{i}\right\}}_{i=1}^{n}$, and triangle sets $T={\left\{t_{j}\right\}}_{j=1}^{m}$ and $\hat{T}={\left\{{\hat{t}}_{j}\right\}}_{j=1}^{m}$, respectively.

**Mean orientation error metric:** The orientation error between the original model and the denoised one can be measured using the mean orientation error metric given by

(20) $$E_{o}=\frac{1}{m}\sum_{j=1}^{m}∠\left(n\left(t_{j}\right),n\left({\hat{t}}_{j}\right)\right),$$

where $n(t_{j})$ and $n({\hat{t}}_{j})$ are the unit face normals of $t_{j}$ and ${\hat{t}}_{j}$, respectively. The symbol, *∠*, denotes the angle between two unit vectors and is defined as the inverse cosine of their dot product.

**Face-normal error metric:** To quantify the performance of the proposed approach, we computed the $L^{2}$ face-normal error metric given by

(21) $$E_{f}\left(M,\hat{M}\right)=\frac{1}{\mathrm{area}(\hat{M})}\sum_{{\hat{t}}_{j}\in \hat{T}}\mathrm{area}\left({\hat{t}}_{j}\right)∥n\left(t_{j}\right)-n\left({\hat{t}}_{j}\right)∥,$$

where $\mathrm{area}({\hat{t}}_{j})$ is the area of ${\hat{t}}_{j}$, and $\mathrm{area}(\hat{M})$ is the total area of the denoised mesh.

**Face quality metric:** The quality of mesh faces can be measured using the ratio of the circumradius-to-minimum edge length given by

(22) $$Q=\frac{1}{\left|T\right|}\sum_{t\in T}\frac{r_{t}}{\ell_{t}},$$

where $r_{t}$ and $\ell_{t}$ are the circumradius and minimum edge length of the associated triangle, respectively. In an ideal case, every face of the mesh should be an equilateral triangle with a quality index equal to $Q=1/\sqrt{3}$.

**Figure 9:**
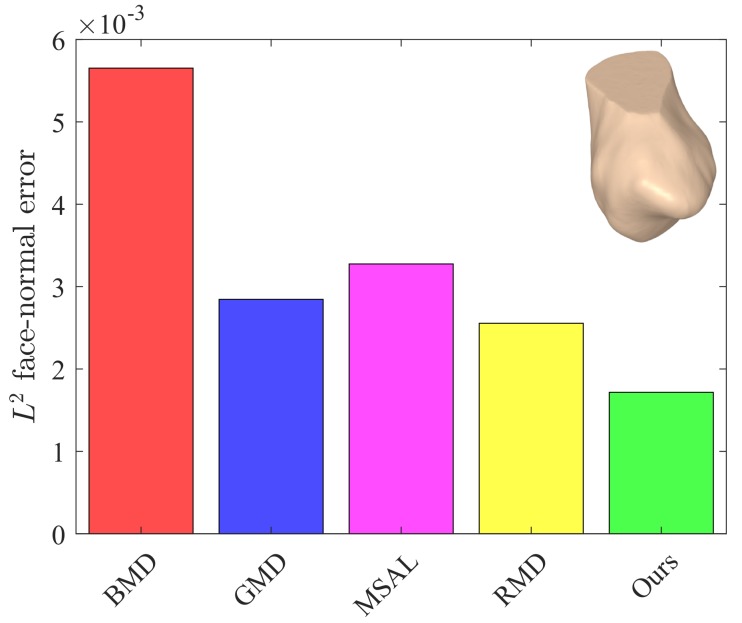
$L^{2}$ face-normal errors for the left metacarpal model.

**Figure 10:**
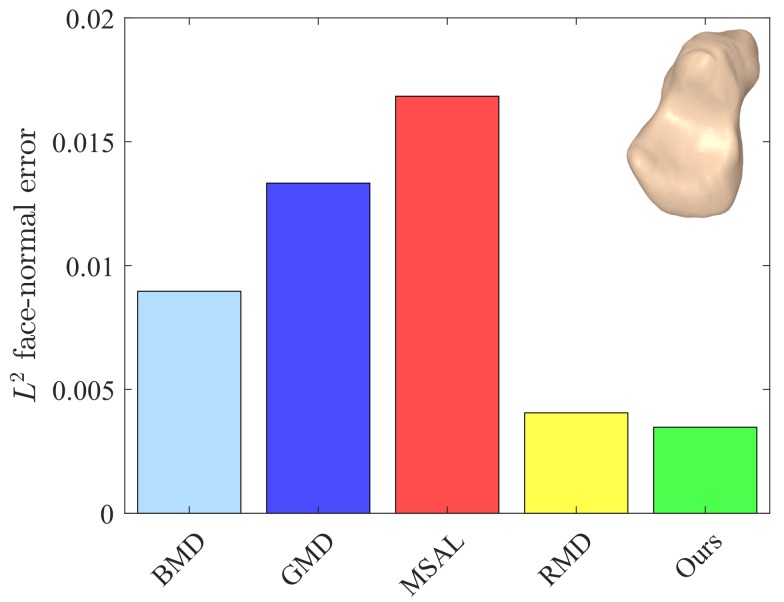
$L^{2}$ face-normal position errors for the scaphoid model.

**Figure 11:**
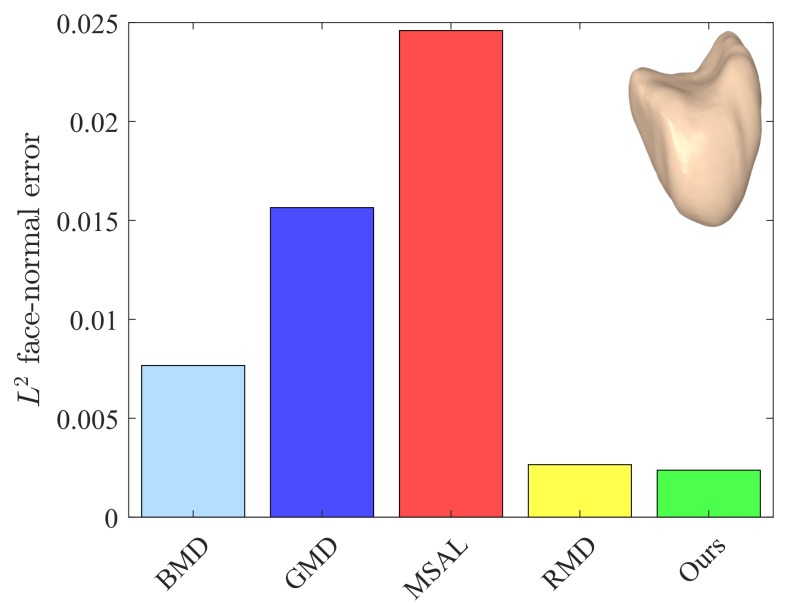
$L^{2}$ face-normal errors for the lunate model.

**Figure 12:**
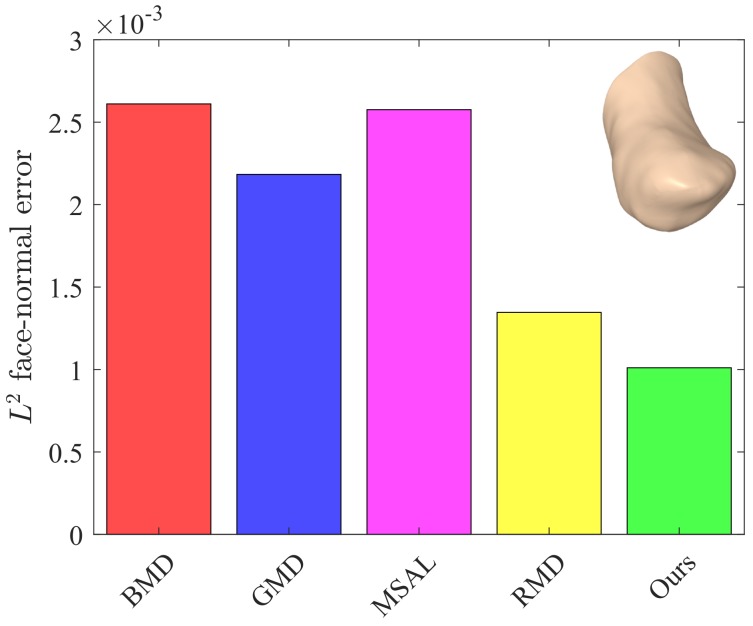
$L^{2}$ face-normal errors for the right metacarpal model.

**Figure 13:**
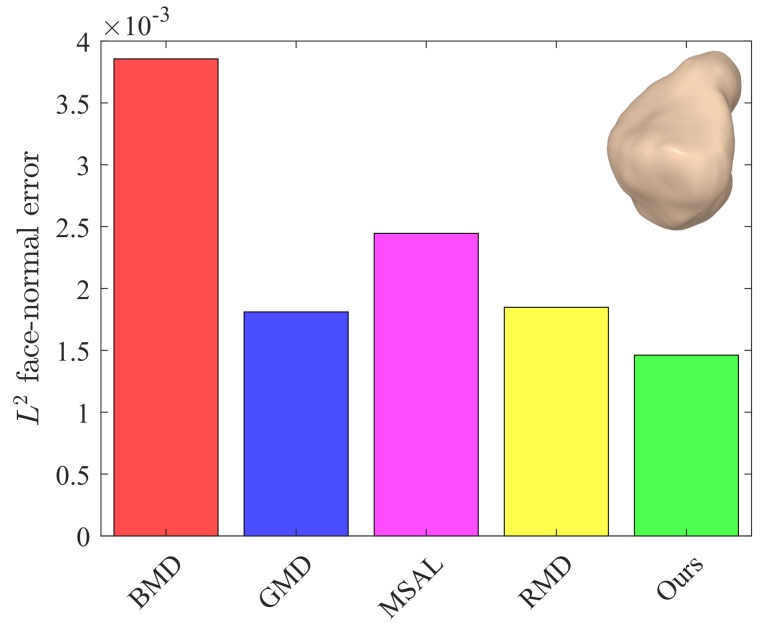
$L^{2}$ face-normal errors for the left hamate model.

The values of these metrics for our approach and the baseline methods are reported in Table 1. For fair comparison, we set the number of iterations to five for all the methods. Our approach yielded better or competitive results in terms of $E_{o}$ and $E_{f}$ for all models. Moreover, the values of *Q* for our method were lower than those of the baseline methods. The $L^{2}$ face-normal errors for the left metacarpal, scaphoid, lunate, right metacarpal, and left hamate are shown graphically in Figure 9, Figure 10, Figure 11, Figure 12 and Figure 13. As can be seen in these figures, our method yielded the best overall results, indicating consistency with the subjective comparison.

#### 4.1.3. Runtime Analysis

Most mesh denoising techniques perform filtering using a two-stage process by first filtering the face normals and then updating the vertex positions to match the filtered face normals, resulting in a computationally expensive process, particularly for large 3D meshes. Our method is, however, fast and simple to implement. Table 2 shows the runtime of our algorithm for different carpal bone models. In comparison, the runtimes (in seconds) per iteration for RMD, which is the best performing baseline method, were 2.555, 2.3004, 2.292 and 2.167 for the right metacarpal, scaphoid, left metacarpal, and left hamate, respectively. This strongly indicates that our algorithm not only performs well in terms of removing undesirable noise from bone surfaces, but is also computationally efficient.

## 5. Conclusions

In this paper, we presented a feature-preserving approach to surface denoising using a data-adaptive similarity in conjunction with matrix balancing. The proposed framework was formulated as a constrained minimization problem. The solution to this problem was estimated iteratively using the conjugate gradient method in an effort to recover sharp features from noisy surfaces. The qualitative and quantitative evaluation results demonstrate that our approach offers superior performance over existing mesh denoising techniques. For the future work, we plan to incorporate edge-aware filters into our framework to tackle data-driven geometry processing problems.

References

## Figures and Tables

**Table 1:** Quantitative comparison results using bilateral mesh denoising (BMD), multiscale anisotropic Laplacian (MSAL), guided mesh normal denoising (GMD), robust and high fidelity mesh denoising (RMD), and our approach.

Model	Method	$E_{o}$	$E_{f}\times 10^{-3}$	*Q*
Right metacarpal |F| = 27,912 |V| = 13,958	BMD	1.503	2.679	2.868
	MSAL	1.506	2.495	6.852
	GMD	1.503	2.183	7.973
	RMD	1.503	2.686	4.700
	Ours	**1.470**	**1.011**	**1.623**
Scaphoid |F| = 29,408 |V| = 14,706	BMD	1.506	7.770	5.226
	MSAL	1.530	16.838	2.247
	GMD	1.457	13.332	7.940
	RMD	**1.453**	3.976	1.888
	Ours	1.465	**2.966**	**1.678**
Left metacarpal |F| = 26,858 |V| = 13,431	BMD	1.510	5.652	2.449
	MSAL	1.512	3.275	1.592
	GMD	1.503	2.845	1.669
	RMD	1.485	2.554	6.404
	Ours	**1.462**	**1.716**	**1.452**
Left hamate |F| = 28,792 |V| = 14,398	BMD	1.494	3.855	13.354
	MSAL	1.506	2.445	5.597
	GMD	**1.386**	1.811	4.663
	RMD	1.418	1.847	2.826
	Ours	1.422	**1.461**	**1.702**

**Table 2:** Runtime (in seconds) per iteration and number of iterations used for denoising different models.

Model	Vertices	Faces	Time (s)	$N_{\mathrm{iter}}$
Right metacarpal	13,958	27,912	0.284	5
Scaphoid	14,706	29,408	0.286	10
Left metacarpal	13,431	26,858	0.487	5
Left hamate	14,398	28,792	0.213	10
